# Composite Graphene-Containing Porous Materials from Carbon for Capacitive Deionization of Water

**DOI:** 10.3390/molecules25112620

**Published:** 2020-06-04

**Authors:** Tamuna Bakhia, Ruslan Kh. Khamizov, Zaur R. Bavizhev, Mukhamed D. Bavizhev, Magomet A. Konov, Daniil A. Kozlov, Snezhana A. Tikhonova, Konstantin I. Maslakov, Matin S. Ashurov, Alexander V. Melezhik, Dmitry A. Kurnosov, Alexander E. Burakov, Aleksey G. Tkachev

**Affiliations:** 1 Department of Chemistry, Lomonosov Moscow State University, 119991 Moscow, Russia; nonvitas@gmail.com; 2 Vernadsky Institute of Geochemistry and Analytical Chemistry of the Russian Academy of Sciences, 119334 Moscow, Russia; khamiz@mail.ru; 3 JSC Scientific Production Enterprise “Radiy”, 125315 Moscow, Russia; zu588@mail.ru (Z.R.B.); mbavizhev@mail.ru (M.D.B.); info@npp-radiy.ru (M.A.K.); 4 Department of Materials Science, Lomonosov Moscow State University, 119991 Moscow, Russia; kozlov@inorg.chem.msu.ru (D.A.K.); kurbatova.snezhana@yandex.ru (S.A.T.); matin_93@mail.ru (M.S.A.); 5 Kurnakov Institute of General and Inorganic Chemistry of the Russian Academy of Sciences, 119071 Moscow, Russia; 6 Nano-Center, Tambov State Technical University, 392000 Tambov, Russia; nanocarbon@rambler.ru (A.V.M.); ozikimoziki@mail.ru (D.A.K.); m-alex1983@yandex.ru (A.E.B.); nanotam@yandex.ru (A.G.T.)

**Keywords:** graphene, aerogel, mesoporous carbon, composite, capacitive deionization

## Abstract

New techniques were developed for the synthesis of monolithic highly porous composite aerogels (hydrogels) from reduced graphene oxide and carbon nanotubes, as well as graphene-containing composites based on mesoporous activated carbon. Simple operations for hydrophilization of synthesized samples were proposed. New electrode materials for electrosorption and deionization of water were fabricated. The resulting materials were investigated and tested in electrochemical cells for membrane capacitive deionization (MCDI).

## 1. Introduction

**Figure 1:**
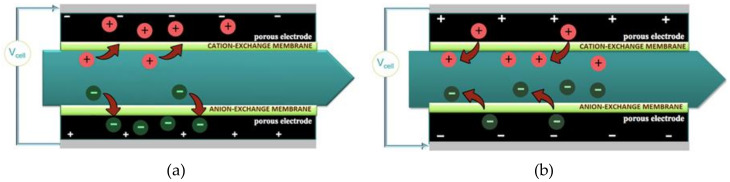
Scheme of membrane capacitive deionization: (**a**) electrosorption and deionization and (**b**) release.

The interest in the synthesis and assembly of carbon nanostructures is associated primarily with the prospects of creating unique macroscopic materials and products from them for practical use in energy, environmental protection, the fabrication of ultra-high and selective sorbents, supercapacitors, and desalination systems of a new generation. Graphene-containing mesoporous composites are auspicious materials for the implementation of new electrochemical technologies. Capacitive deionization of water (CDI), namely, the removal of ions from aquatic solution by applying very small values of an external voltage to electrodes with a large specific surface area, and separate electrosorption of cations and anions, respectively, on the cathode and anode, is today one of the most promising methods of desalination of solutions with low mineralization. The reversibility of the CDI process makes it possible to recover electric energy, and in the future, it will compete with the most economical technologies used today, including reverse osmosis technology [1]. Historical background, experimental approaches to operation, and testing of the CDI are given in the reviews [1,2]. In the CDI method, it is not easy to control the ion desorption process, for example, by reversing the polarity of the voltage on porous electrodes, since the following deionization accompanies the release of ions and the formation of concentrate in the opposite direction. In 2004, the authors from Biosource Inc. [3] patented a method that later became known as membrane capacitive deionization (MCDI) and which allowed the intensification of the desorption stage and increased efficiency and separation ability of cyclic desalination process. A distinctive feature of this variant is that a cation-exchange and anion-exchange membranes are inserted into the electrochemical cell so that they adjoin the electrodes from the inside, as shown in Figure 1. Such a cell operates in deionization mode only at one polarity when the cathode potential is supplied to the upper electrode. When reversing, only the release of ions into the free volume and the formation of concentrate is possible.

The main problem for both variants of capacitive deionization is to obtain accessible electrode materials with high porosity, electrical conductivity, hydrophilicity, and good mechanical properties, allowing the use of many desalination-desorption cycles. Carbon-based aerogels and mesoporous carbon are among the most prospective materials [4]. However, the existing synthesis methods are intended to produce hydrophobic sorption materials, mainly for the collection of oil and oil products [5]. In addition, some methods for aerogels require the use of toxic chemicals like hydrazine for chemical reducing graphene oxide (GO). An environmental friendly technique for producing composite aerogel has been proposed by some of the authors of this article [6], and it allows the use of graphite and multi-walled carbon nanotubes (MWCNTs) as a feedstock and is based on the modified Hummers method for obtaining GO and on some sequence of basic operations, as in [5], but chemical reduction GO is implemented using glucose as a reagent. Microwave radiation is used at different stages of the proposed technique, and it also includes the additional stage of hydrophilization of the final product [6]. Some steps have also been taken to develop methods for the synthesis of mesoporous carbons and to obtain CDI electrode materials from them. Our co-authors from Tambov University have developed an effective method [7], including high-temperature treatment of a mixture of a water-soluble phenol-formaldehyde resin, carbohydrate, and graphene nanoplates. They also have obtained a composite monolithic material, non-destructible in water, using various binders.

This article is devoted to the synthesis and study of new materials for MCDI electrodes with different porosity architectures: highly porous composite aerogels, which is a monolith whose framework is formed, by reduced graphene oxide and nanotubes, as well as the materials on the basis of glued mesoporous carbon micro granules with the addition of graphene.

## 2. Result and Discussion

### 2.1. Synthesis of Composite Aerogels

**Figure 2:**
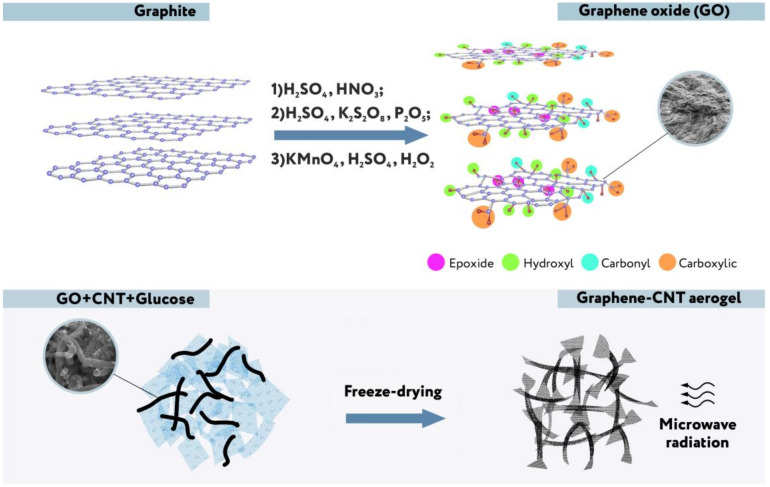
Scheme of some stages of aerogel synthesis.

The sequence of the main stages of our synthesis procedure [6] is presented in Figure 2. A detailed procedure is described in Section 3. There are various modifications of the Hummer’s method based on the intercalation of additional atoms and molecules between molecular layers in graphite in an acidic and oxidizing medium followed by separation of these layers. The procedure used by us at the first stage of the synthesis is closest to the “improved” Hummer’s method proposed in [8]. The resulting graphene oxide (in a mixture of nanotubes), as can be seen in the diagram, undergoes chemical reduction and freeze-drying to obtain a three-dimensional porous structure. At the reducing stage, glucose was used without additional additives. It is a weaker reagent than glucose with ammonia, hydrazine, and some other reducing substances, but creates the possibility of an environmentally friendly process. For the first time, a similar technique for obtaining graphene from graphene oxide (GO) was used in [9]. However, it was known that, besides the reaction of reduction (I) going with the formation of aldonic acid [10], the additional process of functionalization, namely, chemical attachment of glucose residues to the reduced graphene oxide [11], takes place, for example, in accordance with the reaction (II):



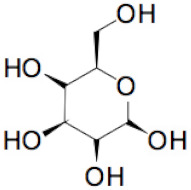

+

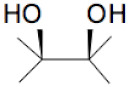

=

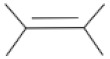

+

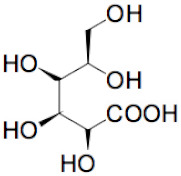

+H_2_O(I)

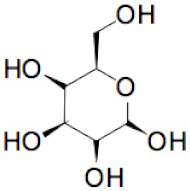

+

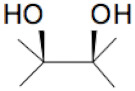

=

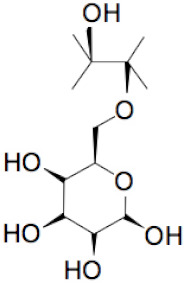

+H_2_O(II)

For the destruction of these organic residues, intensive microwave treatment of resulting aerogel in the form of solid monolith is used before the hydrophilization stage (not shown in Figure 2).

The samples of two types of composite aerogel, “light” and “dense,” were obtained in accordance with the scheme shown. The light sample was more fragile than dense sample. The dense one was synthesized with the use of additional options described in [11]: for a more efficient reduction of graphene oxide by glucose, a metal iron catalyst was applied (see Section 3.1.2). In addition, a dense sample, as will be seen later, had the property of elasticity, to achieve which, after glucose treatment, polyvinyl alcohol was added to the hot reaction mixture. Such an approach was described in [12].

### 2.2. Hydrophilization of Composite Aerogels

**Figure 3:**
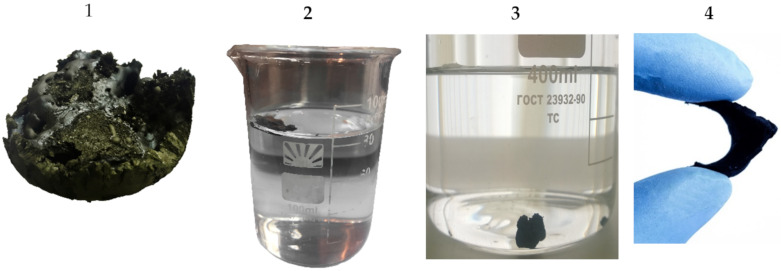
Pictures of aerogel samples obtained: **1**—light sample; **2**,**3**—before and after its hydrophilization; and **4**—denser sample.

The method used for the synthesis of aerogel based on reduced graphene oxide and carbon nanotubes leads to products with hydrophobic properties [5]. Respectively, both samples obtained using the technique described above were not wetted by water and did not drown in it. The products were obtained in the form of monoliths. The denser sample had elastic properties and did not collapse during the multi-faceted bending (Figure 3). After weighing, primary hydrophobic samples or parts of them were used to determine the total density by immersion in water. Then, they were hydrophilized according to the procedure described in the patent [6]: by treatment with a dilute solution of a mixture of nitric acid and hydrogen peroxide. Figure 3 shows the pictures of synthesized samples before and after hydrophilization.

This procedure is essential since hydrophilization involves chemical modification with the sewing of a certain number of functional groups. It is necessary to conduct it so that wettability appears, but at the same time, such an essential characteristic as electrical conductivity does not worsen. Treatment with a dilute solution of hydrogen peroxide and nitric acid, as described above, directed to the results, shown in Table 1.

Here, the density of the initial hydrophobic monolith ($\rho =m/V_{w}$) was calculated as the ratio of the mass of the sample to the volume of displaced water when the sample was immersed in it. For the estimation of porosity, it was taken into account that multilayer graphene forming the skeleton of composite aerogel, should have the same density as graphite: *ρ*_0_ = 2.2 g/cm^3^. In this case, at the porosity ε: $\rho =\varepsilon \rho_{A}+\left(1-\varepsilon \right)\rho_{0}\approx \left(1-\varepsilon \right)\rho_{0}$ where *ρ_A_* = 1.28 · 10^−3^ g/cm^3^—is the density of air from this:

(1) $$\varepsilon \approx 1-\rho /\rho_{0}.$$

### 2.3. Surface and Some Other Properties of Composite Aerogels

**Figure 4:**
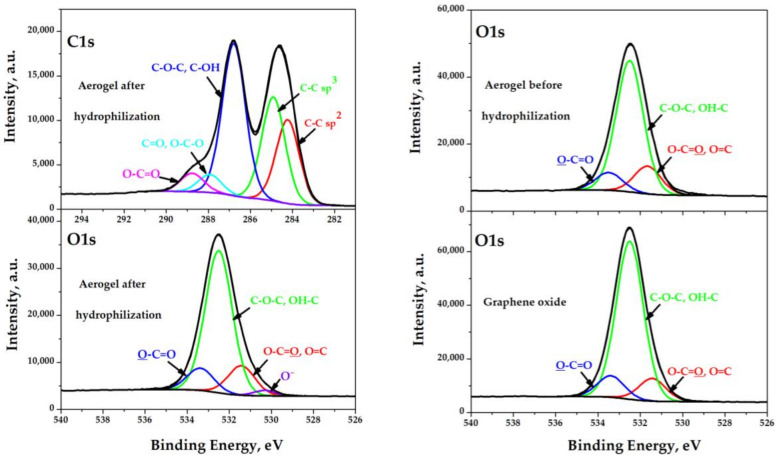
O 1s and C 1s XPS spectra of the samples: graphene oxide and aerogel before and after hydrophilization.

Some of the results presented below explain why the electrical conductivity remained practically unchanged during the hydrophilization of aerogel samples. Figure 4, Table 2 and Table 3 present selected results of X-ray photoelectron spectroscopy, which show the presence of oxygen in the composition of various functional groups and their fragments on the surface of the aerogel before and after hydrophilization. It is seen that the proposed hydrophilization method only leads to a slight functionalization of the surface, which allows us to maintain electrical conductivity, but at the same time, allows us to obtain a sample completely wetted by water.

**Figure 5:**
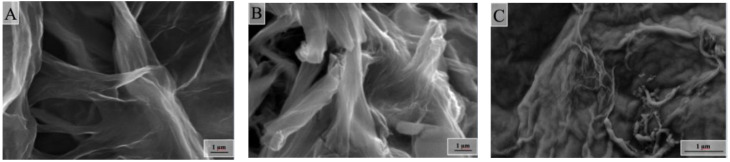
Micrographs (**A**–**C**) of light sample of composite graphene/multi-walled carbon nanotubes (MWCNTs) aerogel at the same magnifications.

It is of great interest to consider the surface morphology of the synthesized samples of composite aerogels. This allows us to judge how the three-dimensional structure of the obtained monoliths is arranged and whether stable porous electrodes can be obtained from them for electrosorption and capacitive deionization. In Figure 5, electron micrographs of the surface of a light sample of a composite aerogel at nearly the same magnifications (with 1 μm) are shown.

One of the main elements of the structure that can be seen in all the images is the differently folded sheets that are connected to each other. Moreover, the patterns shown are almost identical to the microphotographs presented in the article [13].

However, the authors of that article believe that they were able to synthesize a three-dimensional pillar structure in which nanotubes play the role of columns that hold graphene sheets together. It seems to us that three-dimensional structures arise because, during the reduction of graphene oxide (in the course of reducing the effective surface charge), part of the graphene sheets spontaneously folds and chemically sews onto other sheets (or stacks of sheets). This means that the role of the columns is played not by nanotubes, but by rolled sheets of reduced graphene oxide. Multi-walled nanotubes may play some bonding role, but as can be seen in Figure 5C, they are mostly located not across, but along the surfaces of graphene sheets. As shown in Table 3, the assumption that folded sheets can be chemically bonded to each other or other non-folded sheets does not contradict with the results of X-ray photoelectron spectroscopy of the surface of a light hydrophilized sample of a composite aerogel. One can see a corresponding number of carbon atoms included in the C–C bond, with sp^2^ and sp^3^ hybridization for the sample based on reduced graphene oxide.

**Figure 6:**
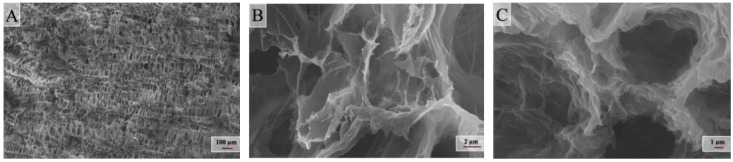
Micrographs of denser sample of composite graphene/MWCNTs aerogel at different magnifications. (**A**): scale bar 100 μm; (**B**): scale bar 2 μm; (**C**): scale bar 1 μm.

Figure 6 shows electron micrographs of the surface of a denser sample of a composite aerogel at different magnifications, and upon close examination of Figure 6B, we can see in its upper-right corner thin filaments of nanotubes, which are also located across the inner surface of the macropore.

**Figure 7:**
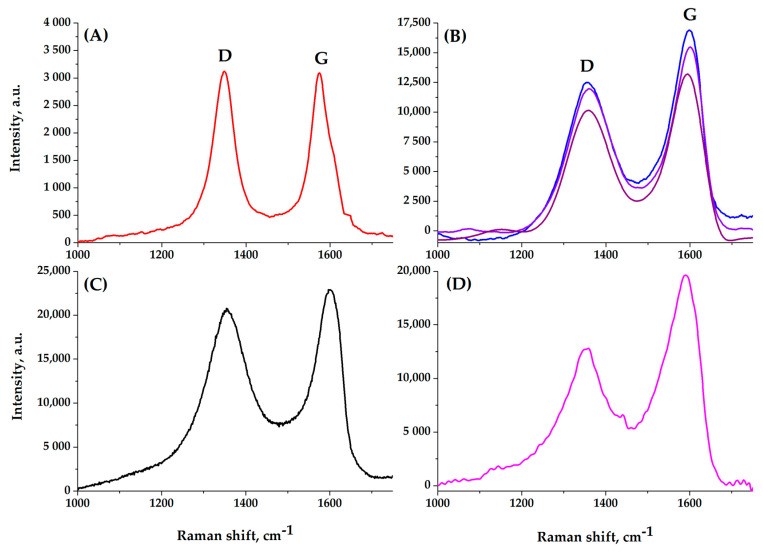
Raman spectra: of initial MWCNTs (**A**), graphene oxide (GO) (**B**) and light (**C**) and denser (**D**) composite aerogel.

Nevertheless, here too, a three-dimensional monolithic structure is created due to reduced graphene oxide. This is consistent with the data in Figure 7, which show the Raman spectra of various samples (after background correction). It can be seen that the reduction of graphene oxide leads only to small changes in the D-peak for carbon atoms. Interestingly, in the spectrum of a light aerogel sample, the D-peak becomes larger than that of GO, in contrast to the spectrum of a dense sample, which shows its decrease. This is due to the fact that during the reduction of graphene oxide we are dealing with two factors: an increase in the number of defects in the structure, which increases sp^3^-hybridization and the reduction process itself, which reduces it. Therefore, with a change in the degree of reduction, the peak ratio first changes in one direction, then in the opposite direction [14]. A significant effect of the chemical interaction of carbon atoms from different layers with a low content of functional groups with oxygen atoms, i.e., the presence of a large number of defects probably also determines the shape of the spectrum for a multi-walled nanotubes shown in Figure 7A. The satisfactory agreement between the spectra of GO samples obtained during various synthesis experiments indicates a good reproducibility of the procedure used.

Let us move from the three-dimensional structure of composite aerogels to their specific porosity. The bulk of the porosity of composite aerogels is formed by macropores, which is clearly seen in Figure 5 and microphotographs of the surfaces shown in Figure 6. One should note that only nanoscale pores (mesopores) are important in the processes of capacitive deionization. Hydrated ions cannot penetrate micropores of atomic sizes. Macropores cannot provide the primary condition for CDI: for that to achieve deionization, it is necessary to fulfill the main requirement: the contents of a solution in the pore can fit on its inner surface. If C—concentration of salt (let it be NaCl) in the solution to be processed, and S—the surface concentration on pore walls, we can roughly estimate the order of pore size to enable capacitive deionization. It is evident for a material balance for a single pore with the diameter d, that:

(2) $$\pi d^{2}S=\frac{1}{6}\pi d^{3}C$$

Y. Oren [1] estimates the surface capacity of carbon materials for salts as about S ≈ 10^−10^ eqv/cm^2^. We can evaluate the order of diameters at which CDI process can be valid at different initial concentration of salt in solution. These estimations are given in Table 4.

A rigorous assessment requires sophisticated modeling, taking into account a large number of factors based on solving the Poisson equation for given boundary conditions and calculating the distribution functions of the potential and component concentrations in the pores [1,15].

**Figure 8:**
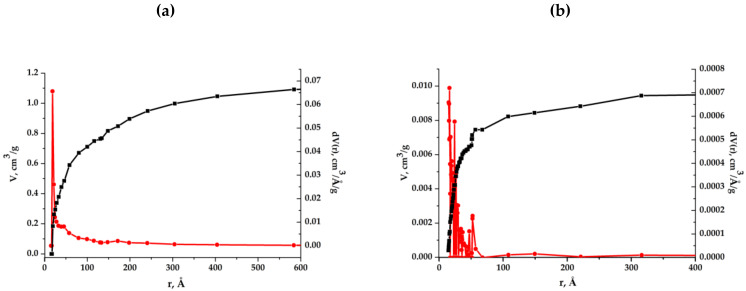
Specific pore volumes and size distribution for light (**a**) and dense (**b**) composite aerogels.

Let us consider the mesopore characteristics for the synthesized samples of composite aerogels found by the sorption (desorption) of nitrogen molecules at low temperature. The results presented in Figure 8 and found with the use of the Barrett–Joyner–Halenda method (BJH) will allow us to see the pore size distribution in the range from atomic sizes to several tens of nm. It can be seen that this distribution has a completely diverse character for two samples of aerogels. In the light sample, some of the pores are formed of the same type with an average size radius of 1 nm, and the other part consists of almost evenly distributed pores of all sizes.

Calculations on the Brunauer–Emmett–Teller method (BET) show that the mesopore surface is around 290 m^2^/g. In the dense sample, mesopores are mainly in the size range of radii of 1–7 nm, the average pore radius = 1.66 nm, the surface area is estimated as 350 m^2^/g.

### 2.4. Organization of MCDI Process

**Figure 9:**
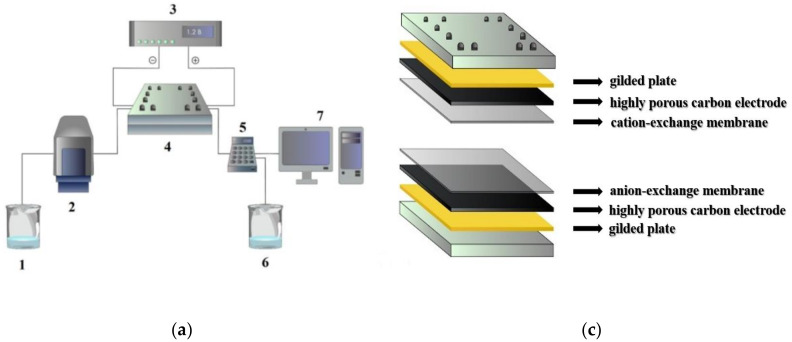

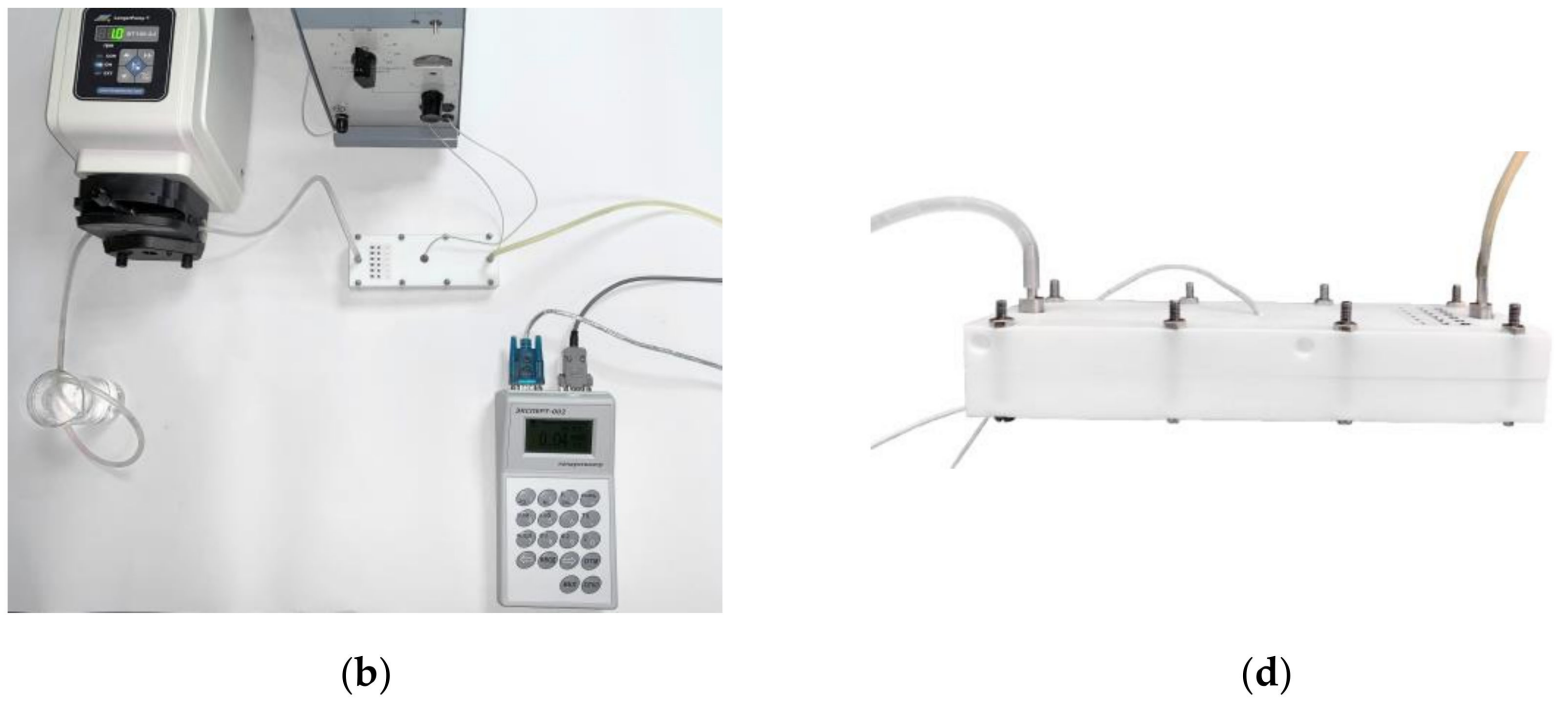
Scheme (**a**) and photo (**b**) of laboratory setup: **1**—salt solution; **2**—peristaltic pump; **3**—potentiostat; **4**—cell; **5**—conductivity; **6**—effluent; and **7**—computer; scheme (**c**) and photo (**d**) of electrochemical cell.

Figure 9 shows a laboratory setup diagram for membrane capacitive deionization, the design of the electrochemical cell, and the corresponding photographs. The experimental result given below were obtained at the following conditions: first, an excess amount of the initial model solution of sodium chloride was passed through the cell in order to ensure equilibrium between it and the phase of the porous electrode. An external DC voltage was then applied to the electrodes to (not more than 1.2 V to avoid electrolysis and the formation of gas bubbles). In this case, the polarity was chosen so that the cathodic potential was applied from the side of the cation-exchange membrane. During a dynamic experiment, one continued to pass the solution through the cell until the electrical conductivity (salt concentration) at the input and output became the same, after which the polarity of the voltage applied to the electrodes was changed. In batch mode, the polarity reversal was done after the minimum conductivity value was reached.

### 2.5. Composite Aerogels in MCDI Process

#### 2.5.1. Results of Experiments with Light Sample

**Figure 10:**
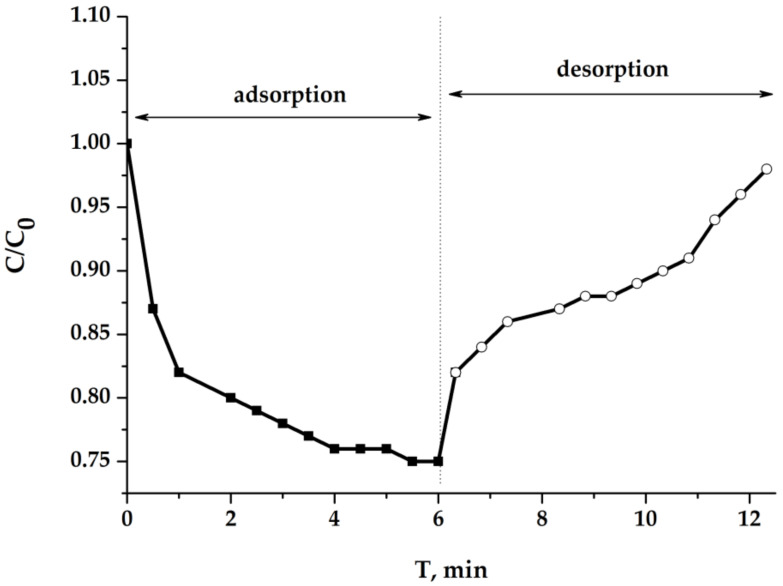
NaCl concentration depending on time in batch process: C_0_ = 1.0 g/dm^3^ (0.017 eqv/dm^3^); U = 1.2 V; porous electrode (cm): 3 × 3 × 0.5; electrode gap: 1 cm.

(It is correct to call the water-containing materials, swollen in an aqueous medium, as the “hydrogels”, but following the literature and in order not to multiply the number of terms, it is advisable to leave the designation “aerogels”). Figure 10 shows the curves of changes in the electrical conductivity of the solution inside a closed electrochemical cell at different stages of the batch mode MCDI process. The ratio of the current and initial electrical conductivity is equal to the ratio of the corresponding salt concentrations. First, the concentration of sodium chloride decreases, and after polarity reversal of the voltage at the electrodes, returns to its original value. The process is completely symmetrical, which indicates the reversibility of electrosorption and desorption. The specific capacity for the sorbed salt (per 1 g of dry electrode material) calculated from these curves is 62 mg/g, and this value, taking into account the equilibrium concentration of salt in a liquid phase, exceeds all known and previously published capacitance values [16,17,18]. We can consider how the same electrodes work in dynamic mode. In order to achieve deeper demineralization, a more diluted stock solution was taken in the experiment. The results obtained are presented in Figure 10.

**Figure 11:**
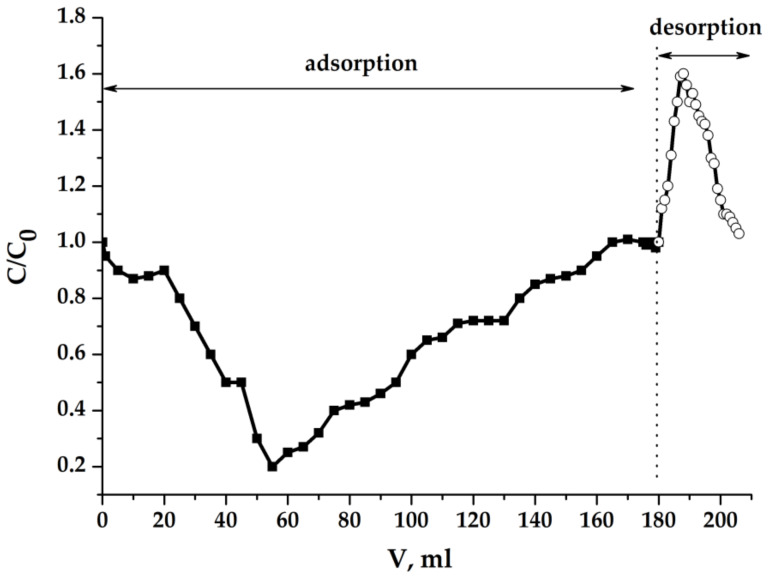
NaCl concentration in the effluent in dynamic process: C_0_(NaCl) = 0.1 g/dm^3^ (0.0717 eqv/dm^3^); U = 1.2 V; porous electrode (cm): 3 × 3 × 0.5; electrode gap: 0.5 cm; flow rate: 0.42 cm^3^/min.

The asymmetric nature of the dynamic curve in Figure 11, in which we see that the amount of salt at the stages of sorption and desorption is different, does not show the irreversibility of the processes in MCDI, but indicates that other type of mass transfer processes can take place simultaneously with them. After the sorption–desorption cycle, we opened the electrochemical cell and saw a significant shrinkage of the porous electrode. This effect can be explained if the slow parallel processes of mass transfer take place, this can be the osmotic transfer of water from such an electrode (whose skeleton is fragile and occupies less than 1% of its total volume) into the inter-electrode space at the stage when desorbed salt accumulates in it. In any case, the obtained results indicate that too much porosity is not always a decisive factor in capacitive deionization.

#### 2.5.2. Results Obtained with the Dense Aerogel Sample

**Figure 12:**
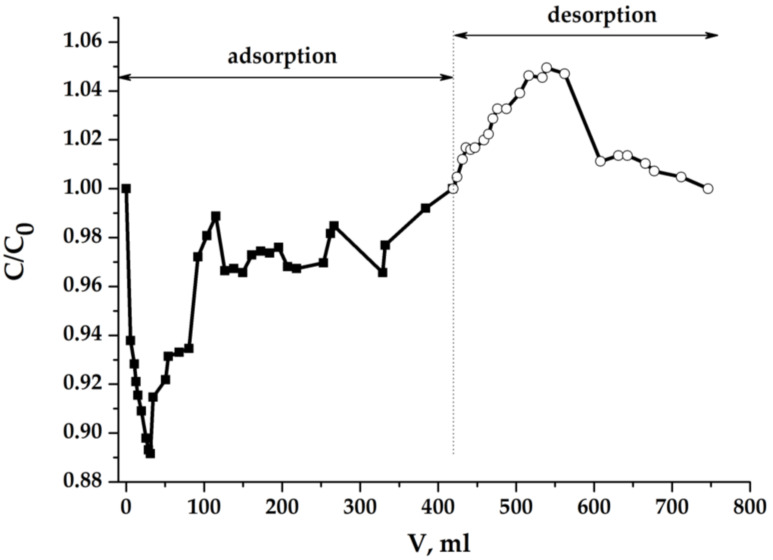
NaCl concentration in the effluent in dynamic process: C_0_ = 1 g/dm^3^ (0.717 eqv/dm^3^); U = 1.2 V; porous electrodes: 3 × 3 × 0.5 cm; electrode gap: 0.5 cm; flow rate: 0.69 cm^3^/min.

Figure 12 shows the curves of changes in the concentration of salt in a solution flow from the electrochemical cell in a dynamic MCDI process with the use of electrodes from the dense aerogel sample.

The process at these electrodes is almost symmetrical. However, it is uneven and generally prolonged (taking into account the flow rate of the solution through the cell). The process becomes faster and more efficient (in terms of desalination depth) if the gap between the electrodes in the electrochemical cell is reduced.

**Figure 13:**
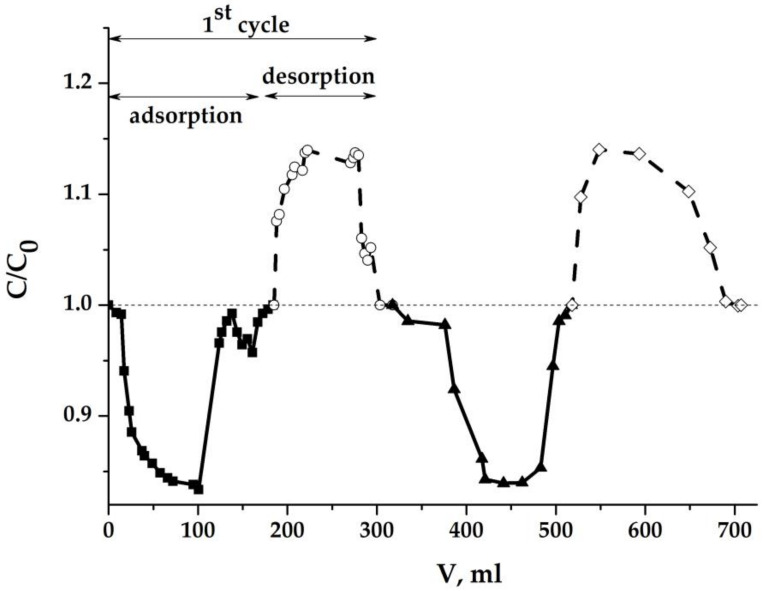
NaCl concentration in the effluent in dynamic process: C_0_ = 1 g/dm^3^ (0.717 eqv/dm^3^); U = 1.2 V; electrodes (cm): 3 × 3 × 0.5; flow rate: 0.69 cm^3^/min; electrode gap: 0.2 cm.

Figure 13 shows the concentration curves for two successive sorption-desorption cycles with a decrease in the gap width to 0.2 cm instead of 0.5. The MCDI process becomes symmetrical and completely reversible and repetitive.

Thus, it can be assumed that when using composite aerogels as electrodes for MCDI under the tested conditions, the diffusion processes in a porous monolith do not limit the rate of deionization of water. The average value of the electrosorption capacity calculated from the experimental data presented in Figure 13 per unit mass of dry aerogel is 25.3 mg/g, which also exceeds that obtained in previously published works [16,18].

### 2.6. Synthesis and Some Properties of Graphene-Containing Mesoporous Carbon (GMC)

The proposed technique [7] for the synthesis of mesoporous activated carbon is based on the process of chemical etching of some organic polymers, especially, phenol–formaldehyde resins (PFR) [19], with the use of KOH at high temperature in an inert atmosphere of nitrogen. A feature of our approach was that graphene was added to the reaction mixture to obtain a graphene-containing composite, and water-soluble PFR with dextrin was also used (see Section 3.2.1).

**Figure 14:**
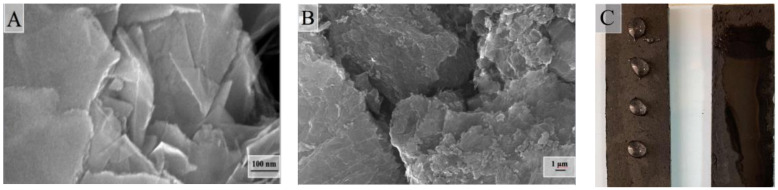
Micrographs of a single granule of mesoporous activated carbon (**A**), the surface of the electrode material (**B**), and photographs of the electrodes before and after hydrophilization (**C**).

Figure 14 represents electron microscopic images of mesoporous carbon on the surface of an electrode obtained from it, as well as the photographs of electrodes with water applied in the form of drops before and after hydrophilization.

It can be seen that the electrode material consists of granules of several dozens of micrometers in size, between which macropores are formed. Mesopores are located inside the granules, but they are not visible at the resolutions of the electron microscope. Unlike the original carbon material and the electrode obtained from it, which are not wetted by water, hydrophilization by simple boiling allows one to obtain electrodes that are instantly permeable with water and on the surface of which water immediately spreads.

**Figure 15:**
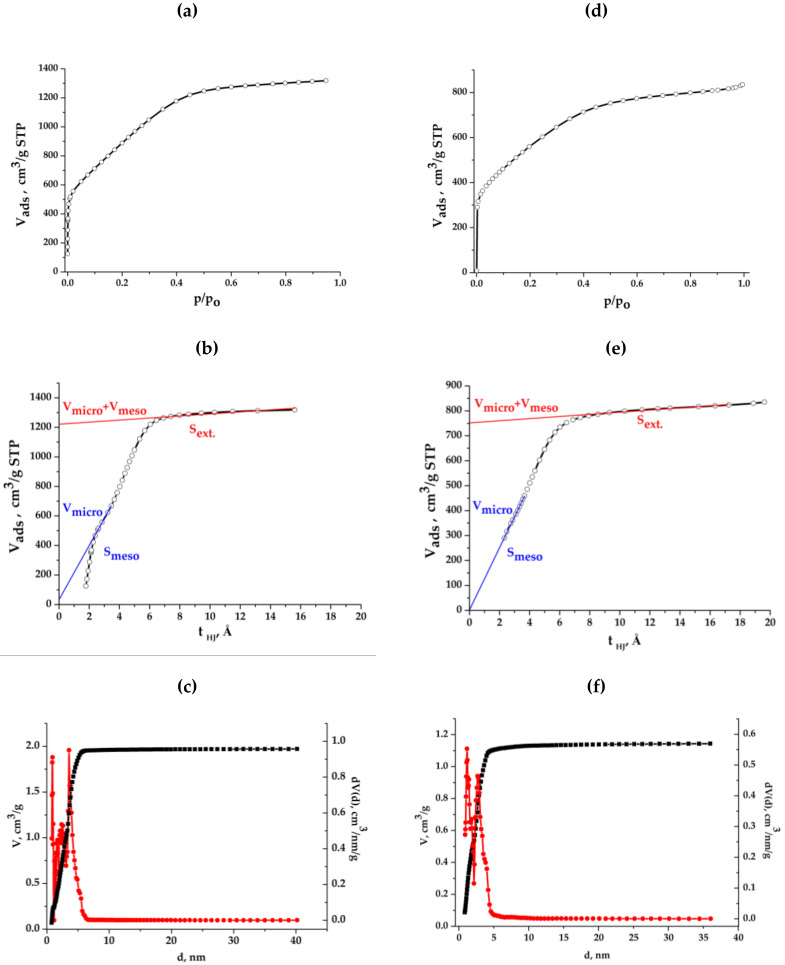
Nitrogen sorption isotherms, t-plot method, pore volume and size distribution (non-local density functional theory (NLDFT) model) for Graphene-Containing Mesoporous Carbon (GMC): activated carbon (**a**–**c**) and the electrode obtained from GMC (**d**–**f**).

The results of porometric studies of resin-derived carbon material—graphene-containing mesoporous activated carbon, are presented in Figure 15. Based on the obtained sorption isotherms for GMC and the electrode obtained from it (Figure 15a,d), the t-plot method of calculations was performed (Figure 15b,e), owing to which it was found that, the bulk of the pore volume is mainly represented by mesopores, and the micropore volume fraction is less than 6% (V_micro + meso_ = 1.9 cm^3^/g; V_micro_ = 0.05 cm^3^/g); the total surface area is 2580 m^2^/g (BET method), and the surface of mesopores: S _meso_ = 1921 m^2^/g, S _micro_ = 450 m^2^/g. It is not so much correct to use a t-plot method for an already glued and then crushed electrode, but taking into account that the micropore area has not decreased, and taking at least 450 m^2^/g, we can roughly estimate: S _BET_ = 1875 m^2^/g and S _ext._ = 200 m^2^/g; S _meso_ ≈ 1225 m^2^/g. In order to draw the correct conclusions about the pore size distribution, we decided to use the non-local density functional theory (NLDFT) method (Figure 15c,f), since it was supposed to study the question of the presence of micropores in the obtained samples. The following data were obtained on the average pore size: a) mesoporous activated carbon—3.55 nm; b) the electrode obtained from GMC—3.1 nm.

The data found with the use of BET method and NLDFT model and presented in Table 5 have been independently obtained by two scientific groups of co-authors. The specific surface around 2000 cm^2^/g indicates that the bulk of the porosity of the studied material is mainly formed by mesopores.

### 2.7. GMC Electrodes in MCDI Process

**Figure 16:**
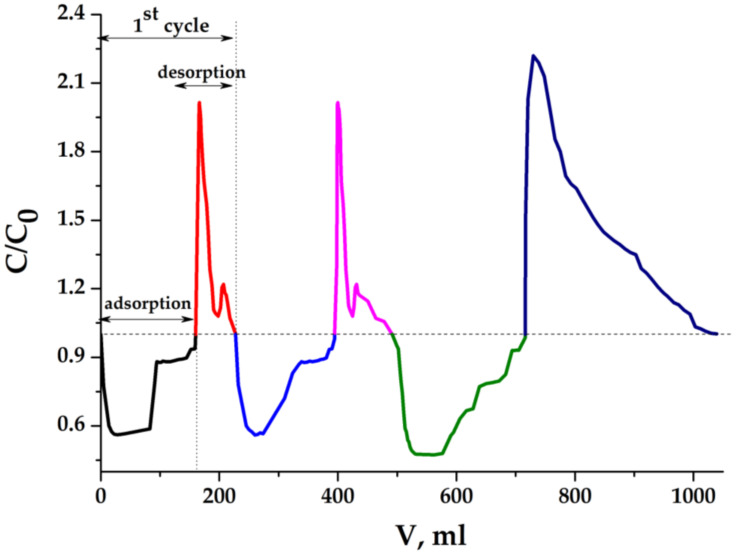
Three successive cycles of membrane capacitive deionization (MCDI) experiments on mesoporous carbon based electrodes: C_0_ = 1 g/dm^3^; U = 1.2 V; electrodes (cm): 12 × 3 × 0.5; electrode gap: 0.3 cm; flow rate: 0.75 cm^3^/min.

Membrane capacitive desalination experiments with glued graphene-containing mesoporous carbon were carried out using larger electrochemical cells with 12 × 3 cm electrodes of different thicknesses. Figure 16 shows the changes in the concentration of the sodium chloride solution flowing from the electrochemical cell in successive sorption-desorption cycles with 0.5 cm thick electrodes. The total amount of salt adsorbed during all three cycles is 240 mg and 245 mg of desorbed salt, which indicates the observance of the material balance, taking into account possible experimental errors.

**Figure 17:**
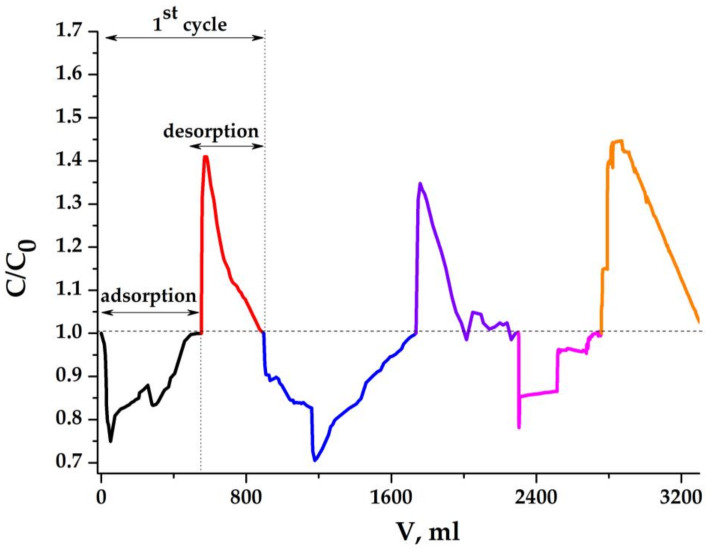
Three successive cycles of MCDI experiments on mesoporous carbon based electrodes C_0_ = 1 g/dm^3^; U = 1.2 V; electrodes (cm): 12 × 3 × 0.2; electrode gap: 0.3 cm; flow rate: 1.15 cm^3^/min.

However, these values are distributed unevenly over the three cycles; desorption in the first two cycles is significantly less than in the third. We suggest that such an effect can occur in the presence of some micropores, and therefore, diffusion difficulties arise in mass transfer, especially at the desorption stage. A small number of micropores, without affecting the electrosorption capacity, can create kinetic difficulties. The most revealing example is the case when a narrow micropore is located between two mesopores along the diffusion flow. It can be imagined that not the entire layer of each of the electrodes works efficiently, but only their inner part is facing the solution. In this case, a deeper layer can be involved in the process at the sorption stage, but sorbed ions can overcome a shorter distance at a slower desorption stage. A non-uniformity may arise since desorption may not end entirely during each polarity reversal period, and the picture shown in the figure is possible. This hypothesis can be verified using the same electrodes, but with a smaller thickness. Figure 17 shows three consecutive cycles of the MCDI process with electrodes that have been ground to a thickness of 0.2 cm.

The flow rate of the sodium chloride solution was also increased. As can be seen from the obtained results, the efficiency (degree) of concentration changes in the processes in different successive sorption-desorption cycles becomes more uniform, although not completely. An increase in velocity leads to the formation of wider peaks at the desorption stages. The total amount of salt adsorbed during all three cycles is 210 mg on average for both stages. This also indicates that the total capacity of electrodes does not substantially change at the decrease of their thickness. The mass of each electrode in the dry state was 2.52 g. Here, we can estimate each electrode’s specific capacity in one cycle of the process: 220:3:5.04 = 14.6 mg/g. The results on MCDI obtained with electrodes made of glued GMC material show that the main limitation in the dynamics of the deionization process is the processes of electro-diffusion transfer in an array of electrodes. One can also agree with the author of article 1 that only a small part of the mesopores works in the MCDI process, adding only: located on the surface of the electrodes facing the solution. This is also evidenced by the fact that the electrodes fabricated from the composite aerogel with a mesopore surface of 350 m^2^/g and from mesoporous activated carbon with a surface of about 1200 m^2^/g show comparable values of the capacity to sodium chloride.

The table below shows the values of the capacitances of electrode materials obtained and investigated in work in comparison with literature data. (The lightweight porous composite aerogel was not taken into account since it cannot be used in real dynamic processes of capacitive deionization).

The data obtained show that the materials synthesized in the presented work and the electrodes obtained from them show the efficiency in the process of capacitive deionization of water, comparable with the best results published in the literature. The specific capacities per volume unit presented in the bottom line were calculated using the density values of dry samples of materials, for example, 0.41 mg/cm^3^ for aerogel in the publication [16].

Our results on similar material are several times better. Very good results on the capacities of several types of composite materials in the CDI process for an initial solution of sodium chloride with an electrical conductivity of 100 μS/cm (which corresponds to approximately 50 mg/dm^3^) are published in the work [18]; however, we cannot compare this data with them given in Table 6. It should be assumed that the new graphene-containing composite materials obtained in this work, namely, aerogels and carbon mesoporous structures, can be useful in other fields, given that such hybrid materials offer great promise for their use in energy and environmental protection [20].

## 3. Materials and Methods

### 3.1. Obtaining Composite Aerogels

Raw materials: dry colloidal graphite preparation from natural graphite, grade S-1 (Russia); multi-walled carbon nanotubes (MWCNTs from “Deatlom”, with inner diameter 2–20 nm, outer diameter 35–100 nm, length (max)—5 μm, electrical conductivity—0.5 mS^−1^); an iron plate (10 × 10 × 1 mm).

Reagents used: KMnO_4_ (p.a.), H_2_SO_4_ (conc., p.a.), HNO_3_ (conc., p.a.), H_2_O_2_ (30%, p.a.), P_2_O_5_ (p.a.), K_2_S_2_O_8_ (p.a.), HCl (conc., p.a.), C_2_H_5_OH (96%), glucose (purissimum), polyvinyl alcohol (purissimum), polyvinylpyrrolidone (with a degree of polymerization around 10,000), as well as deionized water with a specific resistance of at least 1 MΩ·cm.

#### 3.1.1. Light Aerogel Synthesis

The first stage (synthesis of GO). Graphite flakes weighing 5 g were mixed during the day with a mixture of concentrated sulfuric and nitric acids, taken in a volume ratio of 3:1. The mixture was diluted with deionized water and filtered after 2 h. The precipitate was washed with deionized water, dried at 60 °C and treated with microwave radiation (950 W) for 3 min. 1.5 g of the resulting product, 300 cm^3^ of sulfuric acid, 4.2 g of potassium persulfate, 6.2 g of phosphoric anhydride were placed in a flask and stirred for 5 h at a temperature of 80 °C. The mixture was cooled to room temperature, filtered and washed with deionized water. The precipitate was dried. All synthesized powder and 15 g of potassium permanganate were slowly (over an hour) placed in a solution of concentrated sulfuric acid at a temperature 0 °C. The resulting suspension was stirred for two hours at 35 °C, after which it was diluted with deionized water, and then, 10 cm^3^ of hydrogen peroxide was added. The resulting colloidal solution was left overnight. After that, it was centrifuged (5000 rpm) for 20 min, washed with water and 1M hydrochloric acid. The resulting graphene oxide was dispersed in water using ultrasonic treatment. The technique used at this stage is close to that published earlier in works [8,21].

Second stage: 75 mg of MWCNTs, 0.75 mg of polyvinylpyrrolidone, 30 cm^3^ of ethanol were mixed, and the mixture was treated in an ultrasonic bath for 5 h to obtain the colloidal dispersion of carbon nanotubes. After that, this dispersion and the previously dispersed graphene oxide were combined in a mass ratio of 15:1, 0.1 g of glucose was added, the mixture was heated and stirred at 95 °C for an hour, and then, it was left at room temperature for another 2 h.

Third Stage: The mixture was freeze-dried to obtain a monolith aerogel based on a composite of reduced graphene oxide and carbon nanotubes. The synthesized material was treated with microwave radiation for five minutes (950 W/dm^3^).

Fourth Stage: For hydrophilization of the obtained material, it was processed in a solution of nitric acid (5%) and hydrogen peroxide (1%) for 10 min at room temperature.

#### 3.1.2. Dense Aerogel Synthesis

All the above operations were repeated with the exception of the following changes in the second stage: a) an iron plate 10 × 10 × 1 mm in sizes was placed in the colloidal solution in accordance with the method proposed in [11] and b) after the reduction with glucose (in the presence of Fe), 5% by weight of polyvinyl alcohol was added to a colloidal mixture of reduced graphene oxide and carbon nanotubes and stirred for 2 h at a temperature of 60 °C.

Electrodes of the right size were cut manually from the final monolithic materials.

### 3.2. Obtaining Mesoporous Activated Carbon and Electrodes Based on It

Raw materials and Reagents

The following starting materials were used: phenol formaldehyde resin (PFR) of the brand Fenotam GR-326 (LLC Krata, Russia, Tambov), soluble in water with the mass fraction of non-volatile substances 50% at 105 °C; potassium hydroxide (p.a.); graphene nanoplates (GNPs) which were synthesized by ultrasonic dispersion of an expanded graphite compound in an aqueous solution of PFR according to the method described in [22,23] in the form of aqueous paste containing 4.86% graphene and 2.43% PFR as a stabilizer; potato dextrin of premium sort.

#### 3.2.1. Obtaining Mesoporous Activated Carbon

One hundred grams of dextrin and 100 cm^3^ of deionized water were mixed in stainless steel tank (1.5 dm^3^). After swelling for several hours, the mixture was stirred until a uniform, thick solution was formed. 350 g of PFR and 150 g of the aqueous paste of GNPs were added into that solution. The container was closed with a tight-fitting steel cover pressed by springs to prevent free air exchange with the environment. The mixture was heated in an oven at a rate of 10 °C/min with an exposure of 4 h at 140 and 160 °C and 8 h at 300 °C until PFR was solidified. The substance obtained after heat treatment was a solid porous mass, which was then used in parts to prepare the final product.

Then, 10 g of this mass was loaded into a steel beaker for activation and 30 g of potassium hydroxide was added, the beaker was closed with a lid, and the assembly was treated in a muffle furnace with argon duct (0.5 dm^3^/min), temperature was raised at a rate of 5 °C/min to 750 °C, kept at this temperature for 4 h and reaction mixture was quenched with water. The alkaline suspension was filtered, precipitate was washed with water, dried and ground in a mortar. Crushed product was poured with concentrated hydrochloric acid and left for a day to dissolve the impurity of iron. The acid suspension was diluted with water, the product was filtered again, washed with water to a neutral pH and dried in an oven at 110 °C. Then, the obtained mesoporous carbon was subjected to final heat treatment in a stream of argon in a tube furnace at 350 °C.

#### 3.2.2. Fabrication and Hydrophilization of Electrodes

Mesoporous carbon was mixed with fluoroplastic-4 powder in a closed vessel and molded in compliance with the following temperature conditions: melting at a temperature of 375 °C and slow cooling in an argon atmosphere. For hydrophilization of the electrodes they were kept in boiling water for 20 min.

### 3.3. Research Methods Used in the Work

Raman spectroscopy was carried out on a Renishaw InVia Reflex spectrometer/microscope. The survey was conducted on a 514 nm laser (green).

Scanning electron microscopy images were obtained using a Carl Zeiss NVision 40 microscope (3000–300,000×).

X-ray photoelectron spectroscopy spectra were obtained on a Kratos Axis Ultra DLD X-ray photoelectron spectrometer.

The surface area and porosimetry of the samples were determined by capillary sorption of nitrogen on a Quantachrome NOVA 4200e gas sorption analyzer. In this case, two approaches were used: the Brunauer–Emmett–Teller method (BET, determination of the specific surface area), the Barrett–Joyner–Halenda method (BJH, pore size distribution), and the non-local density functional theory (NLDFT) model (pore size distribution).

Study of the electrical conductivity of samples by the 2-pin and 4-pin method (Van der Pauw method). The electrical conductivity was measured in a ProboStat (Norecs) cell by the usual two-electrode method and the van der Pauw method (4-pin). DC conductivity was measured using an Autolab PGSTAT302N/galvanostat potentiostat in cyclic voltammetry (CVC) mode.

The electrical conductivity of the solutions was carried out using an Expert-002-2-6-P conductometer (laboratory sensor).

### 3.4. Materials and Reagents for the Laboratory Setup for MCDI Experiments

Monopolar heterogeneous ion-exchange membranes of brands MK-40 and MA-41 with the thickness in the swollen state of 0.05 cm (LLC Innovative Enterprise Shchekinoazot, Tula, Russia), conductive tape (SPI Supplies, West Chester, PA, USA, 12 mm × 20 m), NaCl (p.a.).

## 4. Conclusions

New techniques are proposed for the synthesis of carbon materials with different porosity architectures: highly porous composite aerogels in monolith form with framework formed by reduced graphene oxide and nanotubes, as well as the graphene-containing materials based on mesoporous activated carbon microgranules. Simple operations for hydrophilization of synthesized samples are proposed. New electrode materials for electrosorption and deionization of water are fabricated, and the resulting materials are investigated and tested in electrochemical cells for membrane capacitive deionization. Superporous (more than 99%) carbon composite aerogels, with a density of not more than 0.02 g/cm^3^, having a rigid structure and characterized by the fragility of the resulting monoliths, are not effective in MCDI deionization processes. Elastic aerogels with a denser structure, porosity of up to 95% and a density of at least 0.12 g/cm^3^ synthesized in the presence of polyvinyl alcohol, have a relatively high capacity to sodium chloride from its solutions of 1 g/dm^3^: up to 25 mg/g (3 mg/cm^3^). They also demonstrate good stability, which makes them very promising for use in capacitive deionization technologies. It assumed that when using composite aerogels as electrodes for MCDI under the tested conditions, the diffusion and electro-diffusion processes in a porous monolith do not limit the rate of deionization of water. Electrodes fabricated from glued graphene-containing mesoporous activated carbon also show excellent properties for their use in electrochemical processes of deionization of solutions. The capacitance values were experimentally obtained: 14.6 mg/g (5.3 mg/cm^3^). The results on MCDI obtained with electrodes made of GMC material show that the main limitation in the dynamics of the deionization process is the processes of electro-diffusion transfer in an array of electrodes. It is approved that only a small part of the mesopores located on the surface of the electrodes facing the solution works in the MCDI process. This is also evidenced by the fact that the electrodes fabricated from the composite aerogel with a mesopore surface of 350 m^2^/g and from mesoporous activated carbon with a surface of about 1200 m^2^/g show comparable values of the capacity to sodium chloride.

## Figures and Tables

**Table 1:** Some primary properties of composite aerogel samples.

Sample	Conductivity before Hydrophilization, *mS/m*	Conductivity after Hydrophilization, *mS/m*	Density of Monolith (*ρ*), g/cm^3^	Porosity, ε∙100%
1 (light)	2000	2000	0.02	99.1
2 (dense)	1900	1900	0.12	95.0

**Table 2:** Oxygen content (in atomic percent) in the composition of functional groups and their fragments on the surface of various samples.

*Samples*	Functional Groups and Fragments			Total *at. %*
	O−C=O ^1^, O=C	C−O−C, OH−C	O ^1^−C=O	
Graphene oxide	3.47	24.05	3.27	30.79
Composite aerogel after hydrophilization	4.32	20.80	3.27	28.39
Composite aerogel before hydrophilization	4.07	19.29	2.63	25.99

**^1^** Underlined symbols indicate the exact atom, which content was determined.

**Table 3:** Element concentrations, binding energies and component fractions for the light sample.

Spectrum	Element Content, *at. %*	Binding Energy, *eV*	Fraction, *at. %*	Type of Bond
**O1s**	29.06	530.3	0.67	O^−^
		531.4	4.32	O−C=O, O=C
		532.5	20.80	C−O−C, OH−C
		533.4	3.27	O−C=O
**C1s**	70.94	284.2	15.77	C−C (sp^2^)
		284.9	19.58	C−C (sp^3^)
		286.8	28.70	C−O−C, C−OH
		287.9	3.40	C=O, O−C−O
		288.7	3.49	O=C−O

**Table 4:** Approximate diameters of mesopores at which the process of capacitive desalination of solutions of different concentrations can be carried out.

*C*, eqv/dm^3^	*C*_NaCl_, g/dm^3^	*d,* nm
**0.01**	0.6	100
**0.1**	5.9	10
**1.0**	58.5	1.0

**Table 5:** Experimental results of physical characteristics of graphene-mesoporous carbon and electrode on its basis.

	Porosity, %	Density, g/cm^3^	Specific Surface Area (S _BET_—S _ext._), m^2^/g	Average Pore Width, nm
GMC	90	0.22	2370	3.55
Electrode	84.2	0.36	1675	3.1

**Table 6:** Specific capacity of electrodes from different materials in capacitive deionization of water (CDI) and MCDI processes (Solution of NaCl = 1000 mg/dm^3^).

Sample	Carbon Aerogel from Activated Fibers [16]	Aerogel MWCNTs-Graphene [17]	MWCNTs Powder [17]	Graphene Powder [17]	Dense Aerogel MWCNTs-Graphene (This Work)	Graphene-Containing Mesoporous Carbon (This Work)
Capacity, mg/g	14.2	24.5	17.5	12.0	25.3	14.6
Capacity, mg/cm^3^	5.7	1.0	-	-	3.0	5.3
